# Antidepressant-Like Effect of Lipid Extract of* Channa striatus* in Chronic Unpredictable Mild Stress Model of Depression in Rats

**DOI:** 10.1155/2016/2986090

**Published:** 2016-12-18

**Authors:** Mohamed Saleem Abdul Shukkoor, Mohamad Taufik Hidayat Bin Baharuldin, Abdul Manan Mat Jais, Mohamad Aris Mohamad Moklas, Sharida Fakurazi

**Affiliations:** ^1^Department of Human Anatomy, Faculty of Medicine and Health Sciences, Universiti Putra Malaysia (UPM), 43400 Serdang, Selangor, Malaysia; ^2^Department of Biomedical Sciences, Faculty of Medicine and Health Sciences, Universiti Putra Malaysia (UPM), 43400 Serdang, Selangor, Malaysia

## Abstract

This study evaluated the antidepressant-like effect of lipid extract of* C. striatus* in chronic unpredictable mild stress (CUMS) model of depression in male rats and its mechanism of action. The animals were subjected to CUMS for six weeks by using variety of stressors. At the end of CUMS protocol, animals were subjected to forced swimming test (FST) and open field test followed by biochemical assay. The CUMS protocol produced depressive-like behavior in rats by decreasing the body weight, decreasing the sucrose preference, and increasing the duration of immobility in FST. The CUMS protocol increased plasma corticosterone and decreased hippocampal and prefrontal cortex levels of monoamines (serotonin, noradrenaline, and dopamine) and brain-derived neurotrophic factor. Further, the CUMS protocol increased interleukin-6 (in hippocampus and prefrontal cortex) and nuclear factor-kappa B (in prefrontal cortex but not in hippocampus). The lipid extract of* C. striatus* (125, 250, and 500 mg/kg) significantly (*p* < 0.05) reversed all the above parameters in rats subjected to CUMS, thus exhibiting antidepressant-like effect. The mechanism was found to be mediated through decrease in plasma corticosterone, increase in serotonin levels in prefrontal cortex, increase in dopamine and noradrenaline levels in hippocampus and prefrontal cortex, increase in BDNF in hippocampus and prefrontal cortex, and decrease in IL-6 and NF-*κ*B in prefrontal cortex.

## 1. Introduction

Acute stress and chronic stress have different effects on human health. Acute stress prepares body for “fight or flight” situation and is beneficial for the survival, while chronic stress may have opposite and deleterious effects [1, 2]. Chronic uncontrolled stress may result in anxiety, depression, and other stress related disorders [3, 4]. Chronic stress in human beings such as psychosocial stress may cause depression in susceptible individuals [5, 6]. External stress factors such as stressful life events and internal stress factors such as chronic inflammation may induce inflammatory, oxidative, and nitrosative stress pathways to precipitate depression in susceptible individuals [7–9]. Chronic stress may suppress the immune system and increases the production of proinflammatory cytokine IL-6 [10]. In animals, the chronic psychosocial stress may induce neuroinflammation and apoptosis and reduced neurogenesis [11].

Chronic stress has an influence on neuroendocrine responses in men [12]. An animal model, chronic unpredictable mild stress (CUMS) model, linking chronic stress and depression was developed by Willner et al. [13, 14]. In the CUMS animal model of depression, the animals are exposed to continuous mild stress such as food deprivation, water deprivation, continuous illumination, tilted cages, and soiled cages in a random unpredictable manner. Usually, after 2 weeks of CUMS, the animals develop variety of symptoms similar to depressive symptoms in human beings such as weight loss, altered diurnal rhythms, sleep disturbances, and anhedonia [13–16]. The anhedonia is reversed by chronic treatment with antidepressants. Several antidepressants showed efficacy in this model [13, 17, 18]. This model mimics the etiology of human depression and has good etiological validity [16, 18].

Chronic uncontrolled stress increases the allostatic load of an organism and impairs homeostasis, thus causing overactivity of some systems and underactivity of some systems in the body [19]. Major depression may be precipitated due to uncontrolled persistent chronic stress [20] due to the induction of inflammatory cytokines [21] in susceptible individuals. There are several disadvantages associated with the current pharmacotherapy for depression. For example, the onset of antidepressant effect takes around 4–6 weeks' time [22, 23] and approximately 30% of patients do not respond at all to currently available drugs [24]. Also, several side effects [25–27] limit the patient compliance to therapy. Hence, development of drugs with better efficacy and safety is required.

*Channa striatus* (called as haruan in Malay) is a freshwater snakehead fish indigenous to Malaysia [28].* C. striatus* belongs to the family Channidae. Its flesh is included in postparturition diet as a rejuvenating diet and to aid wound healing in local Malay population [29]. Aqueous and lipid extracts of* C. striatus* showed significant antidepressant-like effect in our previous experiments [30, 31]. Therefore, we hypothesized that* C. striatus* may have antidepressant-like effect in CUMS model of depression. Hence, this study aimed to evaluate the antidepressant-like effect of* C. striatus* extract in CUMS model of depression and the possible mechanism of action.

## 2. Materials and Methods

### 2.1. Animals

Male Sprague-Dawley rats, approximately 6–9 weeks old, weighing between 150 and 190 g were used. The animals were sourced from Takrif Bistari Enterprise, Seri Kembangan, Selangor, Malaysia. All the animals used in this study were cared for and treated humanely in accordance with the protocols specified by the Institutional Animal Care and Use Committee, UPM, and also with the “Principles of Laboratory Animal Care” (NIH Publication Number 85-23, revised in 1985). The animals were housed for 2 weeks under controlled conditions for acclimatization before the experiments. These conditions were as follows unless otherwise specified: light: 12 h light/dark cycle, lights on at 7:00 am; temperature 25 ± 1°C; free access to food and water. The animals were randomly assigned to different groups for the experiments, namely, no stress (8 animals), CUMS control (7 animals), CUMS + fluoxetine 10 mg/kg (7 animals), CUMS + lipid ext 125 mg/kg (6 animals), CUMS + lipid ext 250 mg/kg (6 animals), CUMS + lipid ext 500 mg/kg (6 animals), and no stress + lipid ext 250 mg/kg (6 animals). All efforts were done to minimize the number of animals used in the experiment. All the experimental protocols were approved by Institutional Animal Care and Use Committee, UPM (UPM/IACUC/AUP-R042/2013).

### 2.2. Chronic Unpredictable Mild Stress (CUMS) Procedure

Chronic unpredictable mild stress was applied in rats for a total duration of 6 weeks based on the previously established protocols [13, 14] with minor modifications as described in previous studies [32, 33]. All animals were subjected to the mild stress protocol in an unpredictable manner for 6 weeks except the animals belonging to no stress + vehicle group and no stress + lipid extract 250 mg/kg group as described in Table 1. The protocol consisted of eight stressors: food deprivation for 24 hr, water deprivation for 24 hr, food and water deprivation for 24 hr, noise for 3 hr (high-pitch medium volume, resembling snake sounds), cage tilting at 45° for 7 hr, overnight illumination for 8 hr, crowded grouped housing (6 rats per cage) for 24 hr, and soiled cage (500 mL water added to 250 g saw dust bedding) for 24 hr. All animals were singly housed except for crowded grouped housing.

The noise and cage tilting stressors were applied randomly at any time of the day. The overnight illumination was carried out from 7.00 pm to 7.00 am the next day. The order of the stressors was randomized in order to avoid any habituation effect. The sucrose preference test was conducted on every Sunday morning between 9 and 10 am. The sucrose preference test requires food and water deprivation 24 hr before the test to avoid any nonspecific influence of diet on sucrose consumption [34]. Hence, food and water deprivation was used as the stressor on all Saturdays. Since food and water deprivation was used on Saturdays, no other stressors were employed on Saturdays. This was done to avoid any effect of the last stressor on the sucrose consumption [34]. The body weight of each animal was noted on every Sunday before the sucrose preference test.

### 2.3. Preparation of Drugs and Administration

**Figure 1:**
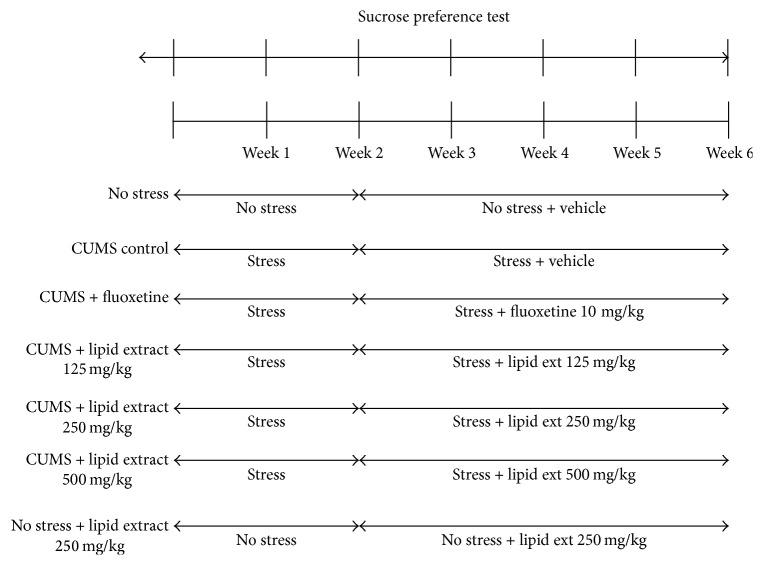
CUMS study scheme and timeline.

The* Channa striatus* (2 kg) was procured from local wet market in Selangor, Malaysia. The fish were identified by Dr. Mohd Shafiq Bin Zakeyuddin, Research Assistant, Department of Environmental Management, Faculty of Environmental Studies, Universiti Putra Malaysia, Malaysia, by following previously published data [35, 36]. A voucher specimen was kept in Human Anatomy Laboratory, Department of Human Anatomy, Faculty of Medicine and Health Sciences, Universiti Putra Malaysia, Malaysia. The fish were killed by a blow to their heads and flesh was separated from bones and fins. The lipid extract was prepared by using the previously published method [37]. The flesh was minced to a paste in a blender. No water was added in this step. The minced paste (800 g) was mixed with 1600 mL (1 : 2 w/v) of chloroform : methanol (2 : 1 v/v) and stirred continuously for 2 hours and filtered. The residue was extracted again with the same solvent mixture at same conditions for another 2 hr and filtered. The filtrates were combined and allowed to stand for 3 hr for separation of aqueous and organic layer. The two layers were separated and evaporated at 40°C in a Rota vapor (Buchi, Switzerland) to remove the solvents separately. The organic extract was dried using lyophilization. The lyophilized extract of organic layer was 80 mL of oily liquid (63.77 g, 7.97% w/w, weight referring to the wet weight of minced fillet paste). Chemical analysis of lipid extract by gas chromatography revealed the presence of oleic acid (23.38%), palmitic acid (18.55%), caprylic acid (16.73%), linoleic acid (10.82%), stearic acid (7.37%), and docosahexaenoic acid (5.85%) as the major constituents with no detected eicosapentaenoic acid (data not shown). Based on the results of our previous experiments (data not shown), three doses (125, 250, and 500 mg/kg) of lipid extract were selected. The lipid extract was oily in nature at room temperature. An emulsion of lipid extract was prepared in normal saline with 5% Tween 80 (Sigma Aldrich, MO, USA) to produce 125, 250, and 500 mg/kg doses. All the drug solutions were prepared fresh on the day of administration. The prepared doses of lipid extract were administered by intraperitoneal route to separate groups of rats after two weeks of CUMS protocol as depicted in Figure 1. The animals which had undergone CUMS protocol showed significant decrease in sucrose preference at the end of 2 weeks as described in Section 3.2. Hence, drug administration was started from week 3 onwards until the sucrose preference was reversed significantly by lipid extract. Fluoxetine (Sigma Aldrich, MO, USA) was used as the positive control and prepared in normal saline with 5% Tween 80 and administered at a dose of 10 mg/kg via intraperitoneal route [38]. One group of animals served as CUMS control group and administered with appropriate vehicle at 10 mL/kg volume via intraperitoneal route. A group of nonstressed animals served as no stress group and administered with appropriate vehicle at 10 mL/kg volume via intraperitoneal route. A group of nonstressed animals were given lipid extract at 250 mg/kg via intraperitoneal route and served as no stress treatment control to assess the effect of lipid extract per se on all the parameters studied. All drug and vehicle administrations were done at a constant volume of 10 mL/kg.

### 2.4. Sucrose Preference Test

The sucrose preference test was conducted based on previously published protocols [13, 34] with minor modifications as described in previous studies [32, 39]. The animals were individually housed in a cage and given two bottles of 1% w/v sucrose solution 72 hr before the actual test on first week Sunday morning. After 24 hr, one bottle containing 1% sucrose solution was replaced with a bottle containing tap water for next 24 hr for the animals to adapt to sucrose solution. After adaptation period, the animals were deprived of food and water for 24 hr. Sucrose preference test was conducted by placing two preweighed bottles to each cage, one containing tap water and the other one containing 1% w/v sucrose solution. The animals had free choice to drink from either bottle. The animals were allowed to drink for 1 hr. The weight of both bottles was recorded and the difference in their respective initial and final weights was calculated. The percentage of sucrose preference was calculated based on the following formula [39]:

(1) %  Sucrose  preference =Sucrose  consumptionSucrose  +  Water  consumption×100

The sucrose preference test was conducted every Sunday morning.

### 2.5. Forced Swimming Test (FST)

The forced swimming test was conducted based on the original method [40] with slight modifications [41]. The apparatus consisted of a plastic cylinder (25 cm diameter × 50 cm height) filled with 30 cm deep water at 24°C ± 2°C. At the end of CUMS protocol, pretest was conducted for 15 min. The animals were individually allowed to swim for 15 min in the swim tank. After the pretest session, the animals were dried with a towel and placed under a heat lamp for 10 min to avoid hypothermia and returned to their respective cages. The water was changed after a trial with each animal to avoid influence to next animal. After 24 hr, same procedure was followed to conduct the test swim session for 5 min. The top view of the activity was recorded with a video camera mounted on the ceiling of the behavior test room. The recorded videos were scored by an observer blind to the treatment regimen and duration of immobility was calculated using a stop watch.

### 2.6. Open Field Test (OFT)

The spontaneous locomotor activity was evaluated by following previously described methods [42]. The apparatus consisted of a square box (75 cm × 75 cm with 42 cm height) made up of plexi glass material. Top of the box was not covered and kept open to observe the movement of the animal. The floor and all sides of the box were covered with white cardboard material. The cardboard at the floor of the box was drawn with black lines dividing the floor into equal squares of 15 cm × 15 cm.

The OFT was conducted at the end of CUMS protocol period on the day of decapitation, before FST. The animals were transferred to OFT test room and acclimatized for 1 hr. The animals were placed onto the center of the box and allowed to explore the box freely for 5 min. After conducting the test on each animal, the box was cleaned with dry tissue paper first and later with 70% alcohol solution and allowed to dry in air to avoid the influence of urine and feces of the previous animal on the next animal. The top view of the box was recorded by a video camera mounted on the ceiling. The video recordings were later scored by an observer blind to the treatment regimen for number of squares crossed and number of rearings. After the OFT, the same animals were tested in FST.

### 2.7. Blood and Brain Tissue Collection and Preparation

At the end of CUMS protocol, 20 min after the FST [43], the animals were decapitated by the use of guillotine. The blood and brain tissue sample preparations were carried out as per the recommendations given in the ELISA kit protocols, Cusabio, Hubei Province, People Republic of China. The trunk blood was collected in an EDTA coated tube and plasma was separated by centrifuging at 1000 ×g at 4°C for 15 min. The plasma was aspirated and used immediately for ELISA analysis or stored at −80°C until analyzed.

The brain was dissected out quickly and carefully and placed on an ice-cold plate. The prefrontal cortex and hippocampus of both sides of the brain were carefully removed [44, 45]. The brain tissues were washed with ice-cold 1x PBS buffer (1 tablet (BR0014G, Oxoid Ltd., UK) dissolved in 100 mL deionized water), weighed, and homogenized in ice-cold 1x PBS solution (100 mg wet tissue in 1 mL 1x PBS) by using Polytron PT-MR 1600 E (Kinematica AG, Switzerland) homogenizer at 1000 rpm for 3 min.

During the homogenization, the Eppendorf tube containing tissue sample was maintained in an ice-cold environment. The homogenates were stored at −20°C overnight and thawed. After two freeze/thaw cycles to break the cell membranes, the homogenates were centrifuged for 5 min at 5000 ×g at 2–8°C. The supernatants were collected and used immediately for ELISA analysis and protein determination or stored at −80°C until analysis. For analysis, either the right or left hippocampus or prefrontal cortex was analyzed. The sample consisted of equal number of right and left hemisphere parts to counterbalance the lateral effects of brain. This procedure was adapted in protein assay as well as in biochemical assays.

### 2.8. Protein Determination

The protein determination was carried out to standardize the expression of results of marker proteins per g of wet brain tissue. Protein concentration was determined in hippocampus and prefrontal cortex tissues of rats by using a protein assay kit from Bio-Rad, CA, USA, with dye reagent concentrate (catalog number 500-0006) and bovine serum albumin as standard (catalog number 500-0002, Bio-Rad). Microassay standard procedure was used. The principle of protein assay is based on Bradford's method [46]. The homogenates from brain tissue were used for protein analysis. A calibration curve was constructed and the unknown concentrations were interpolated and expressed as mg/g of wet brain tissue. Each standard and sample were analyzed in triplicate and mean ± SEM was calculated and used for analysis.

### 2.9. Biochemical Analysis

The plasma was analyzed for corticosterone and oxytocin levels by using ELISA kits as per the manufacturer's instructions (Cusabio, Hubei Province, China). The homogenates from hippocampus and prefrontal cortex were analyzed for serotonin, dopamine, noradrenaline, interleukin-6 (IL-6), nuclear factor-kappa B (NF-*κ*B), and brain-derived neurotrophic factor (BDNF) by using separate enzyme-linked immunosorbent assay (ELISA) kits as per the manufacturer's instructions (Cusabio, Hubei Province, People Republic of China). Briefly, a calibration curve was constructed by using the given standards. Each standard and sample were analyzed in triplicate and mean ± SEM were calculated and used for analysis. The unknown sample concentrations were interpolated from the standard curve and expressed with respect to per gram of protein for IL-6, NF*κ*B, and BDNF.

### 2.10. Statistical Analysis

All the results were expressed as mean ± SEM. The data were analyzed by one-way ANOVA followed by Tukey's multiple comparison test as the post hoc test. All analyses were performed using the software GraphPad Prism version 6.00 for Windows, GraphPad Software, San Diego, California, USA, http://www.graphpad.com/. Effects were considered significant at *p* < 0.05.

## 3. Results

### 3.1. Body Weight

**Figure 2:**
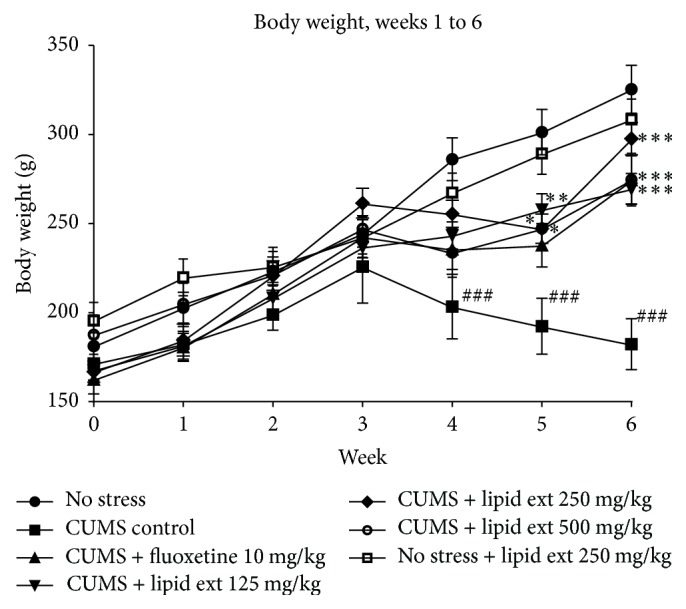
Body weight of rats subjected to chronic unpredictable mild stress. Data represent mean (g) ± SEM (*n* = 6–8); ^###^ *p* < 0.001 when compared to no stress group; ^*∗*^ *p* < 0.05, ^*∗∗*^ *p* < 0.01, and ^*∗∗∗*^ *p* < 0.001 when compared to CUMS control group; one-way ANOVA followed by Tukey's multiple comparison test.

The CUMS control group showed significant reduction in body weight from week 4 onwards when compared to no stress group (Figure 2). At week 5 of CUMS protocol, the body weight of animals increased significantly (*F* = 9.176; df = 6,39; *p* < 0.001) in no stress group, no stress group treated with 250 mg/kg of lipid extract, and groups subjected to CUMS protocol and treated with fluoxetine, 125, 250, and 500 mg/kg doses of lipid extract, when compared to CUMS control group (Figure 2). At week 6, the body weight of all other groups of animals increased significantly (*F* = 14.11; df = 6,39; *p* < 0.001) when compared to CUMS control group (Figure 2). The no stress group which received lipid extract 250 mg/kg did not show any significant change in body weight when compared to no stress group at any of the weeks tested (Figure 2).

### 3.2. Sucrose Preference Test

**Figure 3:**
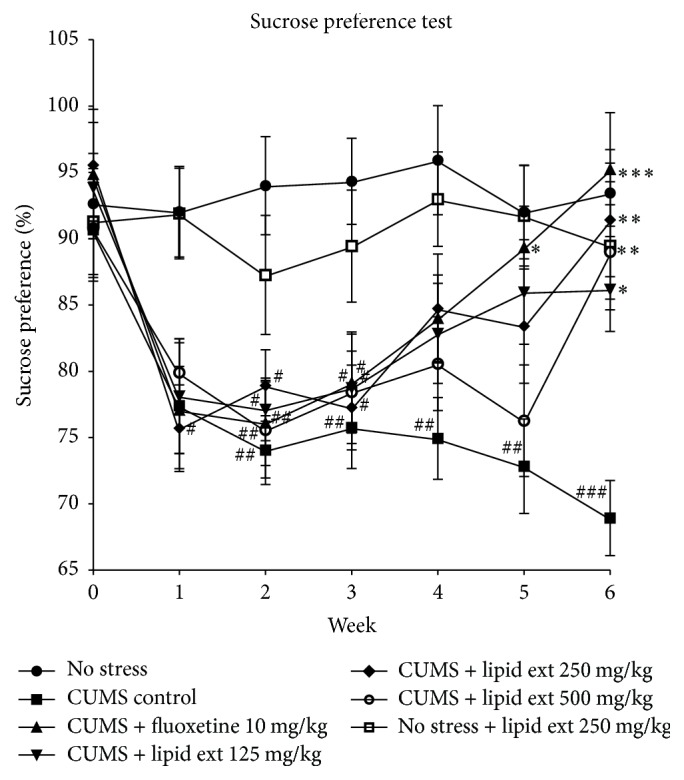
Sucrose preference of rats subjected to chronic unpredictable mild stress. Data represent mean (%) ± SEM (*n* = 6–8); ^#^ *p* < 0.05, ^##^ *p* < 0.01, and ^###^ *p* < 0.001 when compared to no stress group; ^*∗*^ *p* < 0.05, ^*∗∗*^ *p* < 0.01, and ^*∗∗∗*^ *p* < 0.001 when compared to CUMS control group; one-way ANOVA followed by Tukey's multiple comparison test.

At the end of week 2, all animals which had undergone CUMS protocol showed a significant decline (*F* = 5.397; df = 6,39; *p* < 0.001) in their sucrose preference when compared to no stress group (Figure 3). The CUMS control group continued to show significant (*p* < 0.01) decrease in sucrose preference until week 6 (Figure 3). The treatment with lipid extract (125, 250, and 500 mg/kg) significantly (*F* = 5.797; df = 6,39; *p* < 0.001) increased the sucrose preference at week 6 when compared to CUMS control group (Figure 3). The nonstressed animal group which received the lipid extract 250 mg/kg for 6 weeks did not show any significant variation in sucrose preference when compared to no stress group (Figure 3). The treatment with fluoxetine and lipid extract which started in week 3 gradually reversed the sucrose preference almost close to their week 0 baseline levels (Figure 3). The treatment with fluoxetine significantly (*p* < 0.05) increased the sucrose preference at week 5 and week 6 when compared to CUMS control group (Figure 3).

### 3.3. Forced Swimming Test (FST)

**Figure 4:**
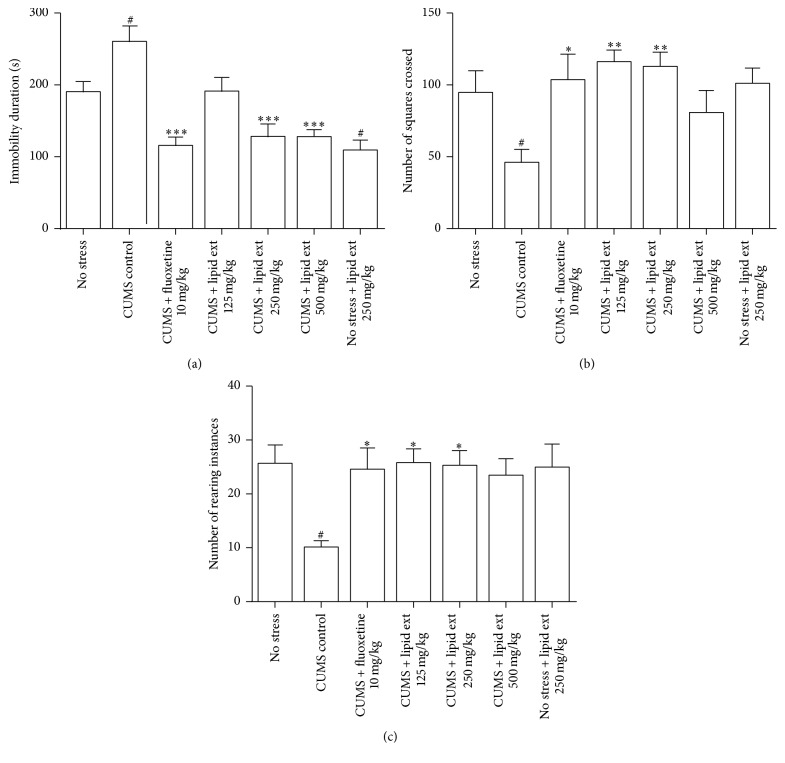
(a) Effect of lipid extract of* C. striatus* fillets and fluoxetine in rats subjected to chronic unpredictable mild stress model of depression in forced swimming test. (b) and (c) Effect of lipid extract of* C. striatus* fillets and fluoxetine in rats subjected to chronic unpredictable mild stress model of depression in open field test. Data represent mean ± SEM (*n* = 6–8). ^#^ *p* < 0.05 when compared with no stress group; ^*∗*^ *p* < 0.05, ^*∗∗*^ *p* < 0.01, and ^*∗∗∗*^ *p* < 0.001 when compared with CUMS control group; one-way ANOVA followed by Tukey's multiple comparison test.

In FST, one-way ANOVA indicated significant difference between the treated groups (*F* = 11.91; df = 6,39; *p* < 0.001). Further post hoc analysis revealed that the CUMS control group had significant (*p* < 0.05) increase in duration of immobility in FST when compared with no stress group (Figure 4(a)). The lipid extract at 250 and 500 mg/kg doses administered to stressed animals produced significant (*p* < 0.001) decrease in the duration of immobility when compared with CUMS control group (Figure 4(a)). Fluoxetine significantly reduced the duration of immobility (*p* < 0.001) when compared to CUMS control group (Figure 4(a)). The animals which were not stressed and administered with lipid extract 250 mg/kg showed significant decrease (*p* < 0.05) in immobility when compared with no stress group.

### 3.4. Open Field Test (OFT)

In OFT, one-way ANOVA indicated significant difference between the treated groups in number of squares crossed (*F* = 3.376; df = 6,39; *p* < 0.01) and in number of rearing instances (*F* = 3.316; df = 6,39; *p* < 0.01). Further post hoc analysis indicated that the CUMS control group had significant (*p* < 0.05) decrease in number of squares crossed (Figure 4(b)) and number of rearing instances (Figure 4(c)) when compared with no stress group. When compared with CUMS control group, the fluoxetine (10 mg/kg) and lipid extract at 125 and 250 mg/kg significantly increased the number of squares crossed (Figure 4(b)) and number of rearing instances (Figure 4(c)). However, when compared to the no stress group, these effects were statistically insignificant.

### 3.5. Biochemical Analysis

#### 3.5.1. Plasma Corticosterone Levels

**Figure 5:**
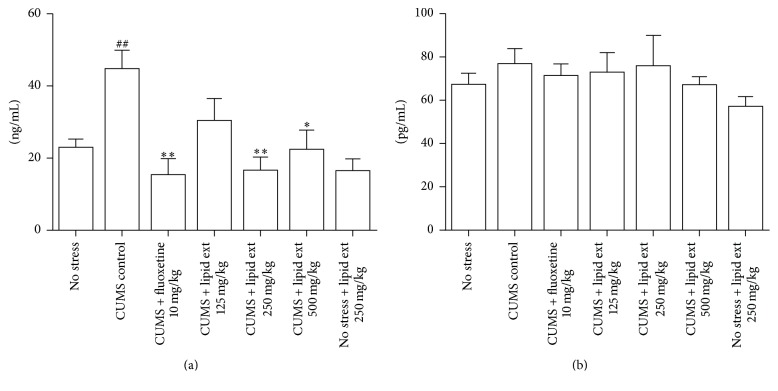
(a) Effect of lipid extract of* C. striatus* fillets and fluoxetine in rats subjected to chronic unpredictable mild stress model of depression on plasma corticosterone level. (b) Effect of lipid extract of* C. striatus* fillets and fluoxetine in rats subjected to chronic unpredictable mild stress model of depression on plasma oxytocin level. Data represent mean ± SEM (*n* = 6–8). ^##^ *p* < 0.05 when compared with no stress group; ^*∗*^ *p* < 0.05 and ^*∗∗*^ *p* < 0.01 when compared with CUMS control group. One-way ANOVA followed by Tukey's multiple comparison test.

The one-way ANOVA indicated significant difference amongst the treated groups (*F* = 5.183; df = 6,39; *p* < 0.001). Further post hoc analysis indicated that the CUMS control group had significant (*p* < 0.01) increase in plasma corticosterone level when compared to no stress group (Figure 5(a)). The fluoxetine significantly (*p* < 0.01) decreased plasma corticosterone level when compared to CUMS control group (Figure 5(a)). The lipid extract (250 and 500 mg/kg) significantly (*p* < 0.05) decreased the plasma corticosterone level in stressed rats when compared to CUMS control group (Figure 5(a)). The nonstressed animals which received lipid extract 250 mg/kg showed no significant change in plasma corticosterone level when compared to no stress group (Figure 5(a)).

#### 3.5.2. Plasma Oxytocin Level

The one-way ANOVA test revealed no significant difference (*F* = 0.6327; df = 6,39; *p* = 0.7032) between all the groups (Figure 5(b)).

#### 3.5.3. Brain Serotonin Levels

**Figure 6:**
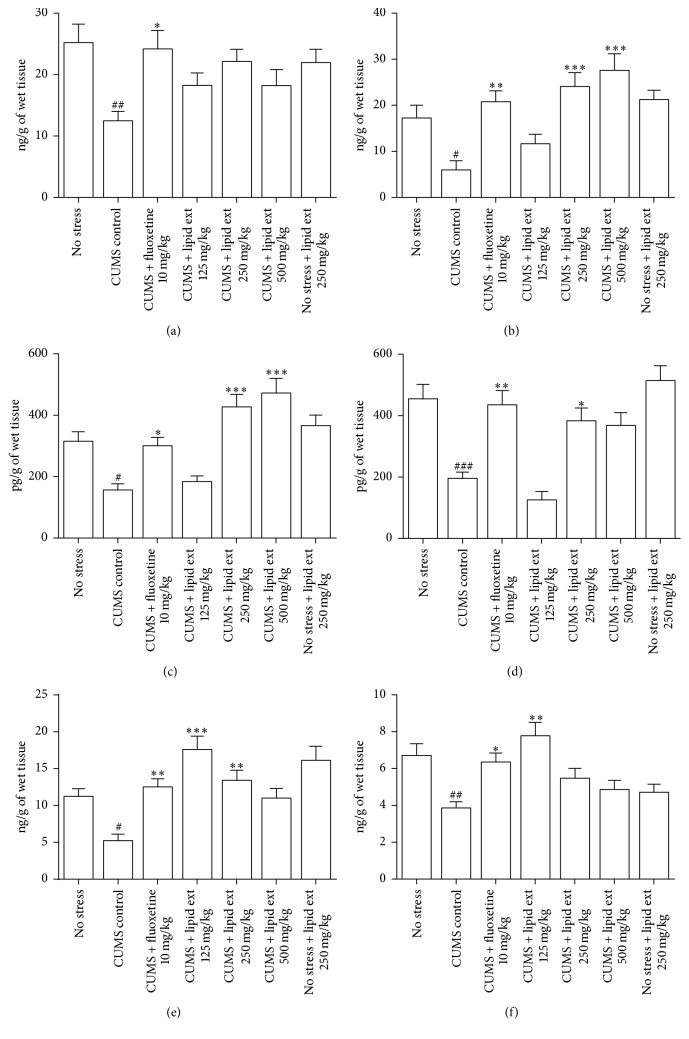
Effect of lipid extract of* C. striatus* fillets and fluoxetine on serotonin level in hippocampus (a), serotonin level in prefrontal cortex (b), noradrenaline level in hippocampus (c), noradrenaline level in prefrontal cortex (d), dopamine level in hippocampus (e), and dopamine level in prefrontal cortex (f) in rats subjected to chronic unpredictable mild stress model of depression. Data represent mean ± SEM (*n* = 6–8). ^#^ *p* < 0.05, ^##^ *p* < 0.01, and ^###^ *p* < 0.001 when compared with no stress group; ^*∗*^ *p* < 0.05, ^*∗∗*^ *p* < 0.01, and ^*∗∗∗*^ *p* < 0.001 when compared with CUMS control group. One-way ANOVA followed by Tukey's multiple comparison test.

The one-way ANOVA test revealed significant difference (*F* = 3.433; df = 6,39; *p* < 0.01) in hippocampal serotonin levels and significant difference (*F* = 7.808; df = 6,39; *p* < 0.001) in prefrontal cortex serotonin levels between the treated groups. In further post hoc analysis, the CUMS control group showed significant (*p* < 0.01) decline in hippocampal (Figure 6(a)) and prefrontal cortex (Figure 6(b)) serotonin levels when compared to no stress group. Fluoxetine significantly (*p* < 0.05) increased serotonin level in hippocampus (Figure 6(a)) and prefrontal cortex (Figure 6(b)) when compared to CUMS control group. The lipid extract produced increase in serotonin level in hippocampus. But, when compared to CUMS control group (Figure 6(a)), the results were statistically insignificant. In contrast, in prefrontal cortex, it significantly (*p* < 0.001) increased serotonin level at 250 and 500 mg/kg when compared to CUMS control group (Figure 6(b)). The nonstressed animals which received lipid extract 250 mg/kg showed no significant change in serotonin level in hippocampus (Figure 6(a)) and prefrontal cortex (Figure 6(b)) when compared to no stress group.

#### 3.5.4. Brain Noradrenaline Levels

The one-way ANOVA test revealed significant difference (*F* = 11.98; df = 6,39; *p* < 0.001) in hippocampal noradrenaline levels and significant difference (*F* = 11.54; df = 6,39; *p* < 0.001) in prefrontal cortex noradrenaline levels between the treated groups. In further post hoc analysis, the CUMS control group showed significant (*p* < 0.05) decline in hippocampal (Figure 6(c)) and prefrontal cortex (Figure 6(d)) noradrenaline level when compared to no stress group. The lipid extract (250 and 500 mg/kg) produced significant (*p* < 0.001) increase in noradrenaline level in hippocampus when compared to CUMS control group (Figure 6(c)). But, in prefrontal cortex, it significantly (*p* < 0.05) increased noradrenaline level at 250 mg/kg dose only when compared to CUMS control group (Figure 6(d)). The nonstressed animals which received lipid extract 250 mg/kg showed no significant change in noradrenaline level in hippocampus (Figure 6(c)) and prefrontal cortex (Figure 6(d)) when compared to no stress group. Fluoxetine significantly (*p* < 0.05) increased noradrenaline level in hippocampus (Figure 6(c)) and prefrontal cortex (Figure 6(d)) when compared to CUMS control group.

#### 3.5.5. Brain Dopamine Levels

The one-way ANOVA test revealed significant difference (*F* = 8.452; df = 6,39; *p* < 0.001) in hippocampal dopamine levels and significant difference (*F* = 5.876; df = 6,39; *p* < 0.001) in prefrontal cortex dopamine levels between the treated groups. In further post hoc analysis, the CUMS control group showed significant (*p* < 0.05) decline in hippocampal (Figure 6(e)) and prefrontal cortex (Figure 6(f)) dopamine levels when compared to no stress group. The lipid extract (125 and 250 mg/kg) produced significant (*p* < 0.01) increase in dopamine level in hippocampus when compared to CUMS control group (Figure 6(e)). But, in prefrontal cortex, it significantly increased dopamine level at 125 mg/kg dose only when compared to CUMS control group (Figure 6(f)). The nonstressed animals which received lipid extract 250 mg/kg showed no significant change in dopamine level in hippocampus (Figure 6(e)) and prefrontal cortex (Figure 6(f)) when compared to no stress group. The fluoxetine significantly (*p* < 0.05) increased dopamine level in hippocampus (Figure 6(e)) and prefrontal cortex (Figure 6(f)) when compared to CUMS control group.

#### 3.5.6. Brain BDNF Level

**Figure 7:**
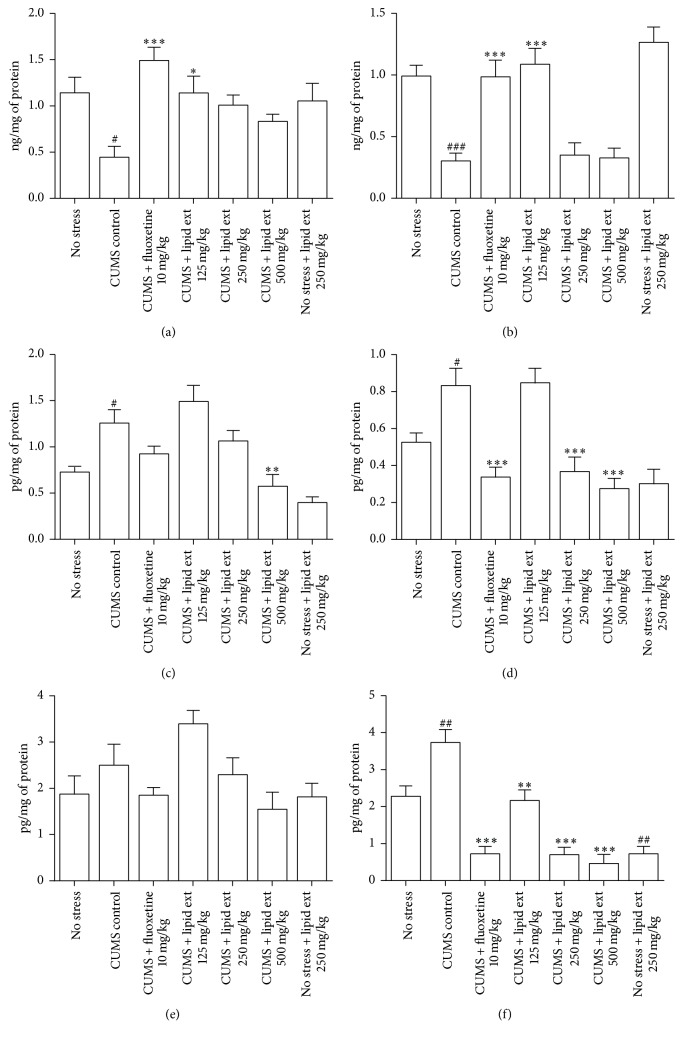
Effect of lipid extract of* C. striatus* fillets and fluoxetine on BDNF level in hippocampus (a) and BDNF level in prefrontal cortex (b), IL-6 level in hippocampus (c), IL-6 level in prefrontal cortex (d), NF-*κ*B level in hippocampus (e), and NF-*κ*B level in prefrontal cortex (f) in rats subjected to chronic unpredictable mild stress model of depression. Data represent mean ± SEM (*n* = 6–8). ^#^ *p* < 0.05, ^##^ *p* < 0.01, and ^###^ *p* < 0.001 when compared with no stress group; ^*∗*^ *p* < 0.05, ^*∗∗*^ *p* < 0.01, and ^*∗∗∗*^ *p* < 0.001 when compared with CUMS control group. One-way ANOVA followed by Tukey's multiple comparison test.

The one-way ANOVA test revealed significant difference (*F* = 4.552; df = 6,39; *p* < 0.01) in hippocampal BDNF levels and significant difference (*F* = 14.76; df = 6,39; *p* < 0.001) in prefrontal cortex BDNF levels between the treated groups. In further post hoc analysis, the CUMS control group showed significant (*p* < 0.05) decline in hippocampal (Figure 7(a)) and prefrontal cortex (Figure 7(b)) BDNF levels when compared to no stress group. The lipid extract (125 mg/kg) produced significant (*p* < 0.05) increase in BDNF level in hippocampus (Figure 7(a)) and prefrontal cortex (Figure 7(b)) when compared to CUMS control group. The nonstressed animals which received lipid extract 250 mg/kg showed no significant change in BDNF level in hippocampus (Figure 7(a)) and prefrontal cortex (Figure 7(b)) when compared to no stress group. The fluoxetine significantly (*p* < 0.001) increased BDNF level in hippocampus (Figure 7(a)) and prefrontal cortex (Figure 7(b)) when compared to CUMS control group.

#### 3.5.7. Brain IL-6 Levels

The one-way ANOVA test revealed significant difference (*F* = 9.537; df = 6,39; *p* < 0.001) in hippocampal IL-6 levels and significant difference (*F* = 11.48; df = 6,39; *p* < 0.001) in prefrontal cortex IL-6 levels between the treated groups. In further post hoc analysis, the CUMS control group showed significant (*p* < 0.001) increase in hippocampal (Figure 7(c)) and prefrontal cortex (Figure 7(d)) IL-6 levels when compared to no stress group. The lipid extract produced significant (*p* < 0.05) decrease in IL-6 levels in hippocampus (Figure 7(c)) at 500 mg/kg and in prefrontal cortex at 250 and 500 mg/kg (Figure 7(d)) when compared to CUMS control group. The nonstressed animals which received lipid extract 250 mg/kg showed no significant change in IL-6 levels in hippocampus (Figure 7(c)) and prefrontal cortex (Figure 7(d)) when compared to no stress group. The fluoxetine did not produce any significant change in IL-6 levels in hippocampus (Figure 7(c)) but significantly (*p* < 0.001) reduced it in prefrontal cortex (Figure 7(d)) when compared to CUMS control group.

#### 3.5.8. Brain NF-*κ*B Levels

The one-way ANOVA test revealed significant difference in hippocampal NF-*κ*B levels (*F* = 2.996; df = 6,39; *p* < 0.05) and in prefrontal cortex NF-*κ*B levels (*F* = 21.46; df = 6,39; *p* < 0.001) between the treated groups. In further post hoc analysis, the CUMS control group showed significant (*p* < 0.01) increase in NF-*κ*B levels in prefrontal cortex (Figure 7(f)) but not in hippocampus (Figure 7(e)) when compared to no stress group. The lipid extract produced no significant decrease in NF-*κ*B levels in hippocampus (Figure 7(e)) but produced highly significant (*p* < 0.001) decrease in NF-*κ*B levels in prefrontal cortex at 125, 250, and 500 mg/kg (Figure 7(f)) when compared to CUMS control group. The nonstressed animals which received lipid extract 250 mg/kg showed no significant change in NF-*κ*B levels in hippocampus (Figure 7(e)) but showed very significant change in NF-*κ*B levels in prefrontal cortex (Figure 7(f)) when compared to no stress group. The fluoxetine did not produce any significant change in NF-*κ*B levels in hippocampus (Figure 7(e)) but significantly (*p* < 0.001) reduced it in prefrontal cortex (Figure 7(f)) when compared to CUMS control group.

## 4. Discussion

### 4.1. Body Weight

The CUMS procedure decreased the body weight of animals similar to the previously reported findings [47, 48]. At week 6, the lipid extract at all doses significantly prevented the decrease in body weight of animals which was induced by CUMS procedure. The nonstressed animals which received lipid extract 250 mg/kg for 6 weeks did not show any significant weight variation when compared with no stress group. The treatment with fluoxetine significantly prevented the decrease in body weight induced by CUMS procedure at week 6, similar to previously reported findings [49, 50]. These results indicate that the CUMS procedure had profound effect on body weight of animals and chronic administration of lipid extract at 250 mg/kg for 6 weeks does not have any significant effect on body weight per se. These results collectively suggest that the lipid extract has a countering effect on chronic unpredictable mild stress.

### 4.2. Sucrose Preference Test

The results of sucrose preference test indicated that the CUMS protocol produced decreased sucrose preference at week 2 in all the rats subjected to CUMS protocol, suggesting anhedonic effect [13, 14]. Anhedonia refers to the decreased ability to carry out or perceive reward-related behaviors [51]. Consumption of sweet solution is a reward-related behavior in animals [15]. The chronic mild stress procedures employed in our study affected that reward-related behavior and produced a depressive-like effect similar to the previously reported findings [13, 15, 17]. Chronic treatment with the antidepressant drug fluoxetine reversed that anhedonic effect, similar to previously reported findings [38, 52]. The chronic treatment with lipid extract (125, 250 and 500 mg/kg) produced gradual and significant reversal of decreased sucrose preference induced by CUMS protocol, at week 6, suggesting an antidepressant-like effect [13, 17]. The nonstressed animal group which received the lipid extract 250 mg/kg dose showed no significant variation in sucrose preference when compared to no stress group, suggesting that the lipid extract at 250 mg/kg per se does not have any significant influence on sucrose consumption by rats.

### 4.3. Forced Swimming Test (FST)

In FST, the CUMS control group exhibited significant increase in duration of immobility suggesting a depressive-like behavior in stressed animals. In previous studies, rats subjected to CUMS protocol exhibited increased duration of immobility in FST [43, 53]. Therefore, our results are similar to the results of previously reported studies. The lipid extract showed significant antidepressant-like effect by reducing the duration of immobility at 250 and 500 mg/kg in stressed rats and at 250 mg/kg in the nonstressed rats. The effect does not appear to be dose-dependent. Fluoxetine showed very significant antidepressant-like effect, similar to previous studies [54, 55]. The effects of lipid extract are comparable to the effect of fluoxetine.

### 4.4. Open Field Test (OFT)

Agents that increase the locomotor activity in open field test, including psychomotor stimulants, convulsants, and anticholinergics, tend to produce a false positive result in FST [56]. Therefore, locomotor activity was assessed in rats in the open field test to rule out any psychomotor stimulant activity [57]. The major difference between the antidepressants and the psychomotor stimulants is that the antidepressants would not cause significant increase in motor activity [58]. The results of our study indicated that the CUMS procedure significantly decreased the exploratory activity in the CUMS control group. Previous studies indicated that the CUMS procedure decreased the exploratory activity in open field test [39, 43, 48]. Furthermore, our results indicated that the treatment with fluoxetine (10 mg/kg) for 4 weeks reversed the decreased exploratory activity induced by CUMS procedure in open field test. A previous study indicated that chronic treatment with fluoxetine at 10 mg/kg in rats successfully reversed the decreased exploratory activity induced by chronic mild stress procedure [59]. Therefore, our results are similar to the previously reported findings. No increased exploratory activity was observed in the open field test in animals treated with lipid extract at all doses, suggesting that the decreased immobility in the FST is not due to any psychomotor stimulant activity, thereby confirming the antidepressant-like effect observed in FST.

### 4.5. Biochemical Analysis

#### 4.5.1. Plasma Corticosterone Levels

The corticosterone is secreted from adrenal cortex in response to adrenocorticotropic hormone released from anterior pituitary gland. Under stressful situations, the secretion of corticosterone is increased. The secretion of corticosterone is highly regulated by its own negative-feedback mechanism [60]. The CUMS control group showed significant increase in plasma corticosterone similar to a previously published study [61].

Our study design included forced swim test at the end of CUMS protocol since chronic mild stress procedure produced depressive-like behavior in FST in a previous study [53]. In our study, after the forced swim test, the animals were sacrificed and blood was collected and analyzed for plasma corticosterone. The forced swim test in rats was reported to produce an increase in corticosterone levels [62]. The CUMS paradigm was also reported to produce increased corticosterone levels in rats [63, 64]. These results suggest that CUMS protocol has significant effect on hypothalamic-pituitary-adrenal axis of rats. Hence, our findings are consistent with the previous findings.

The antidepressant drug fluoxetine attenuated the increase in plasma corticosterone level induced by CUMS paradigm in stressed rats consistent with a previous finding [65]. In our study, the lipid extract (250 and 500 mg/kg) attenuated the increase in plasma corticosterone level induced by CUMS paradigm in stressed rats. These results indicate that the lipid extract has significant effect on hypothalamic-pituitary-adrenal axis, particularly on the plasma level of corticosterone. Further, the results indicated that the effect of lipid extract (250 mg/kg) on plasma corticosterone is comparable to that of fluoxetine.

Previous studies indicate that the *ω*-3 fatty acids have significant effect on the hypothalamic-pituitary-adrenal axis in stressed rats [41, 66]. The lipid extract was found to contain around 5% of docosahexaenoic acid (data not shown). Hence, the role of docosahexaenoic acid in reducing the plasma corticosterone level may be anticipated. Since corticosterone secretion is the end point of hypothalamic-pituitary-adrenal axis, further studies are required to explore the effects of lipid extract on other molecular gateways of hypothalamic-pituitary-adrenal axis.

#### 4.5.2. Plasma Oxytocin Level

Oxytocin has been linked with depression [67]. A previous study indicated the antidepressant-like effect of oxytocin in animals [68]. Therefore, we evaluated plasma oxytocin level in this study. However, a recent study indicated that exposure to chronic mild stress in male rats did not alter the release of plasma oxytocin induced by 8-OH DPAT ((±)-8-Hydroxy-2-(dipropylamino)tetralin) [69]. Similarly, our results also indicated no significant change in plasma oxytocin levels between CUMS control group and no stress group suggesting no significant effect of CUMS on plasma oxytocin level. The lipid extract produced no significant change in oxytocin levels, although a slight decrease was observed in no stress group treated with lipid extract 250 mg/kg. Our study did not analyze central oxytocin level. Hence, further studies are required to know the effect of CUMS protocol and lipid extract on the central oxytocin level.

#### 4.5.3. Brain Monoamine Levels

The involvement of monoamines such as serotonin, noradrenaline, and dopamine in the pathogenesis of depression has been described previously [70, 71]. The existing antidepressants work by increasing the synaptic level of one or more of these neurotransmitters in the brain [70]. Hence, evaluation of monoamines was carried out in this study. The monoamines were evaluated in hippocampus and prefrontal cortex of rats since structural and neurochemical changes in these regions were reported to be associated in the pathogenesis of depression [72–74]. In the present study, chronic mild stress decreased serotonin, noradrenaline, and dopamine in hippocampus and prefrontal cortex, consistent with previously reported findings [38, 64]. These results suggest that chronic mild stress has significant effect on monoaminergic neurotransmission in brain of rats.

Fluoxetine (10 mg/kg) increased the decreased monoamines caused by CUMS protocol in both hippocampus and prefrontal cortex of rats, similar to a previously reported finding [38]. The lipid extract at 250 and 500 mg/kg increased serotonin only in prefrontal cortex and not in hippocampus. This increase was found to be dose-dependent. Similarly, it produced dose-dependent relationship with hippocampal noradrenaline level. However, the lipid extract showed inverse dose-dependent relationship with hippocampal and prefrontal cortex dopamine levels.

These results suggest that the lipid extract of* C. striatus* had significant effect on the brain monoamines and this influence on monoamines may be attributed to its antidepressant-like effect in FST and sucrose preference test consistent with the previously reported findings on fish oils [75, 76]. Furthermore, the nonstressed animals which received lipid extract 250 mg/kg had more or less similar brain monoamine levels when compared to no stress group animals which were also not stressed. This indicates that the lipid extract has complex effect on brain monoamines in rats and the effect depends on whether the rats are stressed or not. In sucrose preference test, the lipid extract at all doses reversed the anhedonia caused by CUMS protocol. Since dopamine is involved in the reward-related behavior [51], our findings suggest the involvement of dopamine in restoring the sucrose preference. The brain monoamine analysis also revealed that the lipid extract increased dopamine levels in prefrontal cortex and hippocampus. Collectively, these findings suggest the link between anhedonia and dopamine and its modulation by lipid extract of* C. striatus*.

The lipid extract was found to contain oleic acid (23.38% w/w) and *α*-linolenic acid (1.10% w/w) (data not shown). Considering the impact of unsaturated fatty acids on the monoaminergic neurotransmission [75, 76], the contribution towards observed antidepressant-like effect of unsaturated fatty acids present in the lipid extract may be anticipated. Further studies are required to assess the metabolite ratio of monoamines and receptor binding in these monoaminergic pathways.

#### 4.5.4. Brain BDNF Level

BDNF is considered as a critical marker and mediator of mood disorders, particularly major depression and assumed to be involved in the etiology, pathogenesis, and treatment response to antidepressants [77, 78]. Chronic administration of antidepressant drugs increases the expression of BDNF in prefrontal cortex and hippocampus of depressed individuals [79]. Therefore, the BDNF levels in hippocampus and prefrontal cortex of rats were studied. Our study results revealed that CUMS protocol reduced the BDNF expression in hippocampus and prefrontal cortex of rats, consistent with previously reported findings [63, 80]. Fluoxetine increased BDNF level in hippocampus and prefrontal cortex similar to a previous study [81].

Fish oil (12% EPA and 18% DHA) supplementation at 3 g/day was reported to produce increased BDNF expression in hippocampus and prefrontal cortex of rats [82]. In rats administered with krill oil (enriched with *ω*-3 fatty acids), upregulation of BDNF was observed in hippocampus [83]. Dietary supplementation with DHA increased pro and mature BDNF levels in rat hippocampus [84]. Similar results were obtained in our study with lipid extract at 125 mg/kg dose which was found to contain 5.85% DHA and 23.38% oleic acid (data not shown). The maximum effect was observed at the lowest dose used in our study. Therefore, our results are consistent with previously reported findings about *ω*-3 fatty acids and supporting the antidepressant-like effect observed in sucrose preference test and FST.

#### 4.5.5. Brain IL-6 Levels

Elevated plasma proinflammatory IL-6 levels were reported in patients with major depression [85, 86]. Evidence indicates that chronic mild stress for 4 weeks in rats produced elevated IL-6 level in hippocampus and cortex of rats [32, 87]. Hence, in this study, we chose to study IL-6 level in hippocampus and prefrontal cortex of rats. In line with the previous reports [32, 87], in our study, CUMS protocol increased the proinflammatory IL-6 in hippocampus and prefrontal cortex. This inflammatory response was effectively countered by the lipid extract of* C. striatus*, especially at 500 mg/kg in both hippocampus and prefrontal cortex, suggesting a potential anti-inflammatory effect of the lipid extract of* C. striatus*. The lipid extract at 125 mg/kg was ineffective in reducing the IL-6 level both in hippocampus and prefrontal cortex. The anti-inflammatory effect of lipid extract appears to be dose-dependent with maximum effect that occurred at maximum dose.

#### 4.5.6. Brain NF-*κ*B Levels

NF-*κ*B is a critical mediator of inflammatory processes [88] and upregulation of NF-*κ*B activity has been observed in chronic stress [89, 90]. Cytokines such as IL-1*β* activate NF-*κ*B signaling to constitute an inflammatory response [91]. NF-*κ*B was found to be involved in the activation of the IL-6 gene [92]. Munhoz et al. reported that chronic unpredictable stress potentiated the increase in NF-*κ*B in the frontal cortex of rats [90]. Hence, it is expected that CUMS paradigm may increase NF-*κ*B level and subsequently IL-6 level. Similar results were obtained in our study. The CUMS protocol increased the NF-*κ*B level in both hippocampus and prefrontal cortex. This suggests that the CUMS protocol induced an inflammatory response in hippocampus and prefrontal cortex of rats. Fluoxetine decreased NF-*κ*B level in hippocampus and prefrontal cortex of rats consistent with previous findings [93, 94]. The lipid extract markedly decreased the NF-*κ*B activation in prefrontal cortex but not significantly in hippocampus. The decrease in the levels of NF-*κ*B in the prefrontal cortex is very striking. Previous studies showed that *ω*-3 fatty acids suppressed the NF-*κ*B activation [95, 96]. Hence, our results are consistent with previously reported findings and suggest an anti-inflammatory effect of lipid extract of* C. striatus*. Furthermore, our results indicated that similar pattern of response was observed in IL-6 and NF-*κ*B protein levels in prefrontal cortex of rats, suggesting a link between IL-6 and NF-*κ*B, as reported previously [92]. Further studies are required to assess the role of IL-6 and NF-*κ*B in rat prefrontal cortex in chronic stress and depression.

Collectively, the mechanisms of action appear to stem from the ability of lipid extract of* C. striatus* to inhibit the activation of NF-*κ*B. Inhibition of NF*κ*B activity might have reduced IL-6 synthesis [92], subsequently leading to decrease in the plasma corticosterone level since IL-6 was reported to activate hypothalamic-pituitary-adrenal axis and increase corticosterone release [97]. Cytokines, and their signaling pathways, were reported to reduce the availability of monoamine neurotransmitters at synaptic cleft by increasing the reuptake of monoamine neurotransmitters in the brain [98, 99]. A recent study by Kong et al. indicated that IL-6 decreased serotonin transporter in JAR cell line and mouse hippocampus [100]. Therefore, with reduction in IL-6 levels, increase in serotonin content at the synaptic cleft (extracellular serotonin) may be expected. Similar effect was observed in our study. The lipid extract at 500 mg/kg inhibited NF-*κ*B in rat prefrontal cortex and subsequently reduced IL-6 in prefrontal cortex and increased serotonin level in prefrontal cortex, thus suggesting that the action of lipid extract may stem from its ability to inhibit NF-*κ*B and IL-6. However, this cannot be concluded from this study. Further studies are required to explore the effect of unsaturated fatty acids in the lipid extract of* C. striatus* on the molecular pathways interconnecting serotonin, NF-*κ*B, and IL-6 in prefrontal cortex of rats.

## 5. Conclusion

This study demonstrated the antidepressant-like effect of lipid extract of* C. striatus* in chronic unpredictable mild stress model of depression in male rats through sucrose preference test and forced swimming test. Furthermore, the mechanism of the observed antidepressant-like effect was found to be mediated through decrease in plasma corticosterone, increase in serotonin levels in prefrontal cortex, increase in dopamine and noradrenaline levels in hippocampus and prefrontal cortex, increase in BDNF in hippocampus and prefrontal cortex, and decrease in IL-6 and NF-*κ*B in prefrontal cortex.

## Figures and Tables

**Table 1:** Schedule of chronic unpredictable mild stress protocol.

Week	Day	Stress protocol	Duration
1	Sunday morning sucrose test		
	Sunday evening	Noise	3 hr
	Monday	Cage tilting	7 hr
	Tuesday	Overnight illumination	12 hr
	Wednesday	Water deprivation	24 hr
	Thursday	Crowded housing	24 hr
	Friday	Soiled cage	24 hr
	Saturday	Food and water deprivation	24 hr

2	Sunday morning sucrose test		
	Sunday evening	Overnight illumination	12 hr
	Monday	Soiled cage	24 hr
	Tuesday	Noise	3 hr
	Wednesday	Food deprivation	24 hr
	Thursday	Cage tilting	7 hr
	Friday	Crowded housing	24 hr
	Saturday	Food and water deprivation	24 hr

3	Sunday morning sucrose test		
	Sunday evening	Noise	3 hr
	Monday	Cage tilting	7 hr
	Tuesday	Soiled cage	24 hr
	Wednesday	Water deprivation	24 hr
	Thursday	Overnight illumination	12 hr
	Friday	Crowded housing	24 hr
	Saturday	Food and water deprivation	24 hr

4	Sunday morning sucrose test		
	Sunday evening	Soiled cage	24 hr
	Monday	Overnight illumination	12 hr
	Tuesday	Food deprivation	24 hr
	Wednesday	Crowded housing	24 hr
	Thursday	Cage tilting	7 hr
	Friday	Noise	3 hr
	Saturday	Food and water deprivation	24 hr

5	Sunday morning sucrose test		
	Sunday evening	Crowded housing	24 hr
	Monday	Cage tilting	7 hr
	Tuesday	Soiled cage	24 hr
	Wednesday	Water deprivation	24 hr
	Thursday	Noise	3 hr
	Friday	Overnight illumination	12 hr
	Saturday	Food and water deprivation	24 hr

6	Sunday morning sucrose test		
	Sunday evening	Noise	3 hr
	Monday	Crowded housing	24 hr
	Tuesday	Food deprivation	24 hr
	Wednesday	Soiled cage	24 hr
	Thursday	Overnight illumination	12 hr
	Friday	Cage tilting	7 hr
	Saturday	Food and water deprivation	24 hr

7	Sunday morning sucrose test, open field test, and forced swim test trial		
	Monday morning forced swim test followed by decapitation		

## References

[1] Gold P. W. (2015). The organization of the stress system and its dysregulation in depressive illness. *Molecular Psychiatry*.

[2] Selye H. (1998). A syndrome produced by diverse nocuous agents. *Journal of Neuropsychiatry and Clinical Neurosciences*.

[3] Miller D. B., O'Callaghan J. P. (2002). Neuroendocrine aspects of the response to stress. *Metabolism: Clinical and Experimental*.

[4] Stetler C., Miller G. E. (2011). Depression and hypothalamic-pituitary-adrenal activation: a quantitative summary of four decades of research. *Psychosomatic Medicine*.

[5] De Kloet E. R., Joëls M., Holsboer F. (2005). Stress and the brain: from adaptation to disease. *Nature Reviews Neuroscience*.

[6] Siegrist J. (2008). Chronic psychosocial stress at work and risk of depression: evidence from prospective studies. *European Archives of Psychiatry and Clinical Neuroscience*.

[7] Maes M. (1995). Evidence for an immune response in major depression: a review and hypothesis. *Progress in Neuro-Psychopharmacology and Biological Psychiatry*.

[8] Maes M. (2008). The cytokine hypothesis of depression: inflammation, oxidative & nitrosative stress (IO&NS) and leaky gut as new targets for adjunctive treatments in depression. *Neuroendocrinology Letters*.

[9] Maes M., Fišar Z., Medina M., Scapagnini G., Nowak G., Berk M. (2012). New drug targets in depression: inflammatory, cell-mediated immune, oxidative and nitrosative stress, mitochondrial, antioxidant, and neuroprogressive pathways. And new drug candidates—Nrf2 activators and GSK-3 inhibitors. *Inflammopharmacology*.

[10] Robles T. F., Glaser R., Kiecolt-Glaser J. K. (2005). Out of balance: a new look at chronic stress, depression, and immunity. *Current Directions in Psychological Science*.

[11] Kubera M., Obuchowicz E., Goehler L., Brzeszcz J., Maes M. (2011). In animal models, psychosocial stress-induced (neuro)inflammation, apoptosis and reduced neurogenesis are associated to the onset of depression. *Progress in Neuro-Psychopharmacology and Biological Psychiatry*.

[12] Matthews K. A., Gump B. B., Owens J. F. (2001). Chronic stress influences cardiovascular and neuroendocrine responses during acute stress and recovery, especially in men. *Health Psychology*.

[13] Willner P., Towell A., Sampson D., Sophokleous S., Muscat R. (1987). Reduction of sucrose preference by chronic unpredictable mild stress, and its restoration by a tricyclic antidepressant. *Psychopharmacology*.

[14] Willner P., Muscat R., Papp M. (1992). Chronic mild stress-induced anhedonia: a realistic animal model of depression. *Neuroscience and Biobehavioral Reviews*.

[15] Papp M., Willner P., Muscat R. (1991). An animal model of anhedonia: attenuation of sucrose consumption and place preference conditioning by chronic unpredictable mild stress. *Psychopharmacology*.

[16] Willner P. (2005). Chronic mild stress (CMS) revisited: consistency and behavioural-neurobiological concordance in the effects of CMS. *Neuropsychobiology*.

[17] Monleon S., Parra A., Simon V. M., Brain P. F., D'Aquila P., Willner P. (1995). Attenuation of sucrose consumption in mice by chronic mild stress and its restoration by imipramine. *Psychopharmacology*.

[18] Willner P. (1997). Validity, reliability and utility of the chronic mild stress model of depression: a 10-year review and evaluation. *Psychopharmacology*.

[19] McEwen B. S. (2004). Protection and damage from acute and chronic stress: allostasis and allostatic overload and relevance to the pathophysiology of psychiatric disorders. *Annals of the New York Academy of Sciences*.

[20] Checkley S. (1996). The neuroendocrinology of depression and chronic stress. *British Medical Bulletin*.

[21] Kiecolt-Glaser J. K., Derry H. M., Fagundes C. P. (2015). Inflammation: depression fans the flames and feasts on the heat. *The American Journal of Psychiatry*.

[22] Delgado P. L. (2004). How antidepressants help depression: mechanisms of action and clinical response. *Journal of Clinical Psychiatry*.

[23] Taylor C., Fricker A. D., Devi L. A., Gomes I. (2005). Mechanisms of action of antidepressants: from neurotransmitter systems to signaling pathways. *Cellular Signalling*.

[24] Crisafulli C., Fabbri C., Porcelli S. (2011). Pharmacogenetics of antidepressants. *Frontiers in Pharmacology*.

[25] Sharma T., Guski L. S., Freund N., Gøtzsche P. C. (2016). Suicidality and aggression during antidepressant treatment: systematic review and meta-analyses based on clinical study reports. *BMJ*.

[26] Masand P. S., Gupta S. (2002). Long-term side effects of newer-generation antidepressants: SSRIS, venlafaxine, nefazodone, bupropion, and mirtazapine. *Annals of Clinical Psychiatry*.

[27] Schweitzer I., Maguire K., Ng C. (2009). Sexual side-effects of contemporary antidepressants: review. *Australian and New Zealand Journal of Psychiatry*.

[28] Mohsin A. K., Ambak M. A. (1983). *Freshwater Fishes of Peninsular Malaysia*.

[29] Wee K. L., Muir (1982). Snakeheads-their biology and culture. *Recent Advances in Aquaculture*.

[30] Saleem A. M., Hidayat M. T., Mat Jais A. M. (2011). Antidepressant-like effect of aqueous extract of Channa striatus fillet in mice models of depression. *European Review for Medical and Pharmacological Sciences*.

[31] Saleem A. M., Taufik Hidayat M., Jais A. M. M. (2013). Involvement of monoaminergic system in the antidepressant-like effect of aqueous extract of Channa striatus in mice. *European Review for Medical and Pharmacological Sciences*.

[32] Jiang H., Wang Z., Wang Y. (2013). Antidepressant-like effects of curcumin in chronic mild stress of rats: involvement of its anti-inflammatory action. *Progress in Neuro-Psychopharmacology and Biological Psychiatry*.

[33] Lin P.-Y., Chang A. Y. W., Lin T.-K. (2014). Simvastatin treatment exerts antidepressant-like effect in rats exposed to chronic mild stress. *Pharmacology Biochemistry and Behavior*.

[34] Papp M. (2012). Models of affective illness: chronic mild stress in the rat. *Current Protocols in Pharmacology Chapter 5:Unit 5.9*.

[35] Ambak M. A., Isa M. M., Zakaria M. Z., Ghaffar M. A. (2010). *Fishes of Malaysia*.

[36] Rainboth W. J. (1996). *FAO Species Identification Field Guide for Fishery Purposes. Fishes of the Cambodian Mekong*.

[37] Folch J., Lees M., Stanley G. H. S. (1957). A simple method for the isolation and purification of total lipides from animal tissues. *The Journal of Biological Chemistry*.

[38] Yu Y., Wang R., Chen C. (2013). Antidepressant-like effect of trans-resveratrol in chronic stress model: behavioral and neurochemical evidences. *Journal of Psychiatric Research*.

[39] Su G. Y., Yang J. Y., Wang F. (2014). Antidepressant-like effects of Xiaochaihutang in a rat model of chronic unpredictable mild stress. *Journal of Ethnopharmacology*.

[40] Porsolt R. D., Anton G., Blavet N., Jalfre M. (1978). Behavioural despair in rats: a new model sensitive to antidepressant treatments. *European Journal of Pharmacology*.

[41] Arbabi L., Baharuldin M. T. H., Moklas M. A. M., Fakurazi S., Muhammad S. I. (2014). Antidepressant-like effects of omega-3 fatty acids in postpartum model of depression in rats. *Behavioural Brain Research*.

[42] Prut L., Belzung C. (2003). The open field as a paradigm to measure the effects of drugs on anxiety-like behaviors: a review. *European Journal of Pharmacology*.

[43] Dalla C., Antoniou K., Drossopoulou G. (2005). Chronic mild stress impact: are females more vulnerable?. *Neuroscience*.

[44] Chiu K., Lau W. M., Lau H. T., So K.-F., Chang R. C.-C. (2007). Micro-dissection of rat brain for RNA or protein extraction from specific brain region. *Journal of Visualized Experiments*.

[45] Spijker S., Li K. W. (2011). Dissection of rodent brain regions. *Neuroproteomics*.

[46] Bradford M. M. (1976). A rapid and sensitive method for the quantitation of microgram quantities of protein utilizing the principle of protein-dye binding. *Analytical Biochemistry*.

[47] Willner P., Moreau J.-L., Nielsen C. K., Papp M., Sluzewska A. (1996). Decreased hedonic responsiveness following chronic mild stress is not secondary to loss of body weight. *Physiology and Behavior*.

[48] Luo D. D., An S. C., Zhang X. (2008). Involvement of hippocampal serotonin and neuropeptide Y in depression induced by chronic unpredicted mild stress. *Brain Research Bulletin*.

[49] Zhao J., Jung Y.-H., Jang C.-G., Chun K.-H., Kwon S. W., Lee J. (2015). Metabolomic identification of biochemical changes induced by fluoxetine and imipramine in a chronic mild stress mouse model of depression. *Scientific Reports*.

[50] Chen C., Wang L., Rong X., Wang W., Wang X. (2015). Effects of fluoxetine on protein expression of potassium ion channels in the brain of chronic mild stress rats. *Acta Pharmaceutica Sinica B*.

[51] Gorwood P. (2008). Neurobiological mechanisms of anhedonia. *Dialogues in Clinical Neuroscience*.

[52] Deng X.-Y., Li H.-Y., Chen J.-J. (2015). Thymol produces an antidepressant-like effect in a chronic unpredictable mild stress model of depression in mice. *Behavioural Brain Research*.

[53] Kompagne H., Bárdos G., Szénási G., Gacsályi I., Hársing L. G., Lévay G. (2008). Chronic mild stress generates clear depressive but ambiguous anxiety-like behaviour in rats. *Behavioural Brain Research*.

[54] Dang H., Chen Y., Liu X. (2009). Antidepressant effects of ginseng total saponins in the forced swimming test and chronic mild stress models of depression. *Progress in Neuro-Psychopharmacology and Biological Psychiatry*.

[55] Qi X., Lin W., Li J. (2008). Fluoxetine increases the activity of the ERK-CREB signal system and alleviates the depressive-like behavior in rats exposed to chronic forced swim stress. *Neurobiology of Disease*.

[56] Bourin M., Fiocco A. J., Clenet F. (2001). How valuable are animal models in defining antidepressant activity?. *Human Psychopharmacology*.

[57] Walsh R. N., Cummins R. A. (1976). The open-field test: a critical review. *Psychological Bulletin*.

[58] Borsini F., Meli A. (1988). Is the forced swimming test a suitable model for revealing antidepressant activity?. *Psychopharmacology*.

[59] Rong H., Wang G., Liu T., Wang H., Wan Q., Weng S. (2010). Chronic mild stress induces fluoxetine-reversible decreases in hippocampal and cerebrospinal fluid levels of the neurotrophic factor S100B and its specific receptor. *International Journal of Molecular Sciences*.

[60] Herman J. P., Cullinan W. E. (1997). Neurocircuitry of stress: central control of the hypothalamo-pituitary-adrenocortical axis. *Trends in Neurosciences*.

[61] Ayensu W. K., Pucilowski O., Mason G. A., Overstreet D. H., Rezvani A. H., Janowsky D. S. (1995). Effects of chronic mild stress on serum complement activity, saccharin preference, and corticosterone levels in Flinders lines of rats. *Physiology & Behavior*.

[62] Drossopoulou G., Antoniou K., Kitraki E. (2004). Sex differences in behavioral, neurochemical and neuroendocrine effects induced by the forced swim test in rats. *Neuroscience*.

[63] Zhang Y., Gu F., Chen J., Dong W. (2010). Chronic antidepressant administration alleviates frontal and hippocampal BDNF deficits in CUMS rat. *Brain Research*.

[64] Sheikh N., Ahmad A., Siripurapu K. B., Kuchibhotla V. K., Singh S., Palit G. (2007). Effect of *Bacopa monniera* on stress induced changes in plasma corticosterone and brain monoamines in rats. *Journal of Ethnopharmacology*.

[65] Li S., Wang C., Wang M., Li W., Matsumoto K., Tang Y. (2007). Antidepressant like effects of piperine in chronic mild stress treated mice and its possible mechanisms. *Life Sciences*.

[66] Ferraz A. C., Delattre A. M., Almendra R. G. (2011). Chronic *ω*-3 fatty acids supplementation promotes beneficial effects on anxiety, cognitive and depressive-like behaviors in rats subjected to a restraint stress protocol. *Behavioural Brain Research*.

[67] Purba J. S., Hoogendijk W. J. G., Hofman M. A., Swaab D. F. (1996). Increased number of vasopressin- and oxytocin-expressing neurons in the paraventricular nucleus of the hypothalamus in depression. *Archives of General Psychiatry*.

[68] Arletti R., Bertolini A. (1987). Oxytocin acts as an antidepressant in two animal models of depression. *Life Sciences*.

[69] Grippo A. J., Sullivan N. R., Damjanoska K. J. (2005). Chronic mild stress induces behavioral and physiological changes, and may alter serotonin 1A receptor function, in male and cycling female rats. *Psychopharmacology*.

[70] Elhwuegi A. S. (2004). Central monoamines and their role in major depression. *Progress in Neuro-Psychopharmacology and Biological Psychiatry*.

[71] Krishnan V., Nestler E. J. (2008). The molecular neurobiology of depression. *Nature*.

[72] Sapolsky R. M. (2001). Depression, antidepressants, and the shrinking hippocampus. *Proceedings of the National Academy of Sciences of the United States of America*.

[73] George M. S., Ketter T. A., Post R. M. (1994). Prefrontal cortex dysfunction in clinical depression. *Depression*.

[74] Drevets W. C., Price J. L., Furey M. L. (2008). Brain structural and functional abnormalities in mood disorders: implications for neurocircuitry models of depression. *Brain Structure and Function*.

[75] Chalon S., Delion-Vancassel S., Belzung C. (1998). Dietary fish oil affects monoaminergic neurotransmission and behavior in rats. *Journal of Nutrition*.

[76] Chalon S. (2006). Omega-3 fatty acids and monoamine neurotransmission. *Prostaglandins, Leukotrienes and Essential Fatty Acids*.

[77] Hashimoto K., Shimizu E., Iyo M. (2004). Critical role of brain-derived neurotrophic factor in mood disorders. *Brain Research Reviews*.

[78] Castrén E., Rantamäki T. (2010). Role of brain-derived neurotrophic factor in the aetiology of depression: implications for pharmacological treatment. *CNS Drugs*.

[79] Duman R. S., Monteggia L. M. (2006). A neurotrophic model for stress-related mood disorders. *Biological Psychiatry*.

[80] Xu Y., Ku B., Tie L. (2006). Curcumin reverses the effects of chronic stress on behavior, the HPA axis, BDNF expression and phosphorylation of CREB. *Brain Research*.

[81] Molteni R., Calabrese F., Bedogni F. (2006). Chronic treatment with fluoxetine up-regulates cellular BDNF mRNA expression in rat dopaminergic regions. *International Journal of Neuropsychopharmacology*.

[82] Vines A., Delattre A. M., Lima M. M. S. (2012). The role of 5-HT_ 1A_ receptors in fish oil-mediated increased BDNF expression in the rat hippocampus and cortex: a possible antidepressant mechanism. *Neuropharmacology*.

[83] Wibrand K., Berge K., Messaoudi M. (2013). Enhanced cognitive function and antidepressant-like effects after krill oil supplementation in rats. *Lipids in Health and Disease*.

[84] Wu A., Ying Z., Gomez-Pinilla F. (2008). Docosahexaenoic acid dietary supplementation enhances the effects of exercise on synaptic plasticity and cognition. *Neuroscience*.

[85] Liu Y., Ho R. C.-M., Mak A. (2012). Interleukin (IL)-6, tumour necrosis factor alpha (TNF-*α*) and soluble interleukin-2 receptors (sIL-2R) are elevated in patients with major depressive disorder: a meta-analysis and meta-regression. *Journal of Affective Disorders*.

[86] Sukoff Rizzo S. J., Neal S. J., Hughes Z. A. (2012). Evidence for sustained elevation of IL-6 in the CNS as a key contributor of depressive-like phenotypes. *Translational Psychiatry*.

[87] You Z., Luo C., Zhang W. (2011). Pro- and anti-inflammatory cytokines expression in rat's brain and spleen exposed to chronic mild stress: involvement in depression. *Behavioural Brain Research*.

[88] Karin M., Delhase M. (2000). The I*κ*B kinase (IKK) and NF-*κ*B: key elements of proinflammatory signalling. *Seminars in Immunology*.

[89] Koo J. W., Russo S. J., Ferguson D., Nestler E. J., Duman R. S. (2010). Nuclear factor-*κ*B is a critical mediator of stress-impaired neurogenesis and depressive behavior. *Proceedings of the National Academy of Sciences of the United States of America*.

[90] Munhoz C. D., Lepsch L. B., Kawamoto E. M. (2006). Chronic unpredictable stress exacerbates lipopolysaccharide-induced activation of nuclear factor-*κ*B in the frontal cortex and hippocampus via glucocorticoid secretion. *The Journal of Neuroscience*.

[91] Miller A. H., Maletic V., Raison C. L. (2009). Inflammation and its discontents: the role of cytokines in the pathophysiology of major depression. *Biological Psychiatry*.

[92] Libermann T. A., Baltimore D. (1990). Activation of interleukin-6 gene expression through the NF-*κ*B transcription factor. *Molecular and Cellular Biology*.

[93] Liu D., Wang Z., Liu S., Wang F., Zhao S., Hao A. (2011). Anti-inflammatory effects of fluoxetine in lipopolysaccharide(LPS)-stimulated microglial cells. *Neuropharmacology*.

[94] Lim C.-M., Kim S.-W., Park J.-Y., Kim C., Yoon S. H., Lee J.-K. (2009). Fluoxetine affords robust neuroprotection in the postischemic brain via its anti-inflammatory effect. *Journal of Neuroscience Research*.

[95] Novak T. E., Babcock T. A., Jho D. H., Helton W. S., Espat N. J. (2003). NF-*κ*B inhibition by *ω*-3 fatty acids modulates LPS-stimulated macrophage TNF-*α*-transcription. *American Journal of Physiology—Lung Cellular and Molecular Physiology*.

[96] Zúñiga J., Cancino M., Medina F. (2011). N-3 PUFA supplementation triggers PPAR-*α* activation and PPAR-*α*/NF-*κ*B interaction: anti-inflammatory implications in liver ischemia-reperfusion injury. *PLoS ONE*.

[97] Raber J., O'Shea R. D., Bloom F. E., Campbell I. L. (1997). Modulation of hypothalamic-pituitary-adrenal function by transgenic expression of interleukin-6 in the CNS of mice. *Journal of Neuroscience*.

[98] Morón J. A., Zakharova I., Ferrer J. V. (2003). Mitogen-activated protein kinase regulates dopamine transporter surface expression and dopamine transport capacity. *Journal of Neuroscience*.

[99] Zhu C.-B., Blakely R. D., Hewlett W. A. (2006). The proinflammatory cytokines interleukin-1beta and tumor necrosis factor-alpha activate serotonin transporters. *Neuropsychopharmacology*.

[100] Kong E., Sucic S., Monje F. J. (2015). STAT3 controls IL6-dependent regulation of serotonin transporter function and depression-like behavior. *Scientific Reports*.

